# Global trends in the production and use of DDT for control of malaria and other vector-borne diseases

**DOI:** 10.1186/s12936-017-2050-2

**Published:** 2017-10-05

**Authors:** Henk van den Berg, Gamini Manuweera, Flemming Konradsen

**Affiliations:** 1 Laboratory of Entomology, Wageningen University, P.O. Box 8031, 6700EH Wageningen, The Netherlands; 2 Secretariat of Basel, Rotterdam and Stockholm Conventions (BRS), United Nations Environment Programme, Geneva, Switzerland; 3 Department of Public Health, University of Copenhagen, Copenhagen, Denmark

**Keywords:** Malaria, Leishmaniasis, Insecticide resistance, Vector control

## Abstract

**Background:**

DDT was among the initial persistent organic pollutants listed under the Stockholm Convention and continues to be used for control of malaria and other vector-borne diseases in accordance with its provisions on acceptable purposes. Trends in the production and use of DDT were evaluated over the period 2001–2014.

**Results:**

Available data on global production of DDT showed a 32% decline over the reporting period, from 5144 to 3491 metric tons of active ingredient p.a. Similarly, global use of DDT, for control of malaria and leishmaniasis, showed a 30% decline over the period 2001–2014, from 5388 metric tons p.a. to 3772 metric tons p.a. India has been by far the largest producer and user of DDT. In some countries, DDT is used in response to the development of resistance in malaria vectors against pyrethroid and carbamate insecticides. Some other countries have stopped using DDT, in compliance to the Convention, or in response to DDT resistance in malaria vectors. Progress has been made in establishing or amending national legal measures on DDT, with the majority of countries reportedly having measures in place that prohibit, or restrict, the production, import, export and use of DDT. Limitations in achieving the objectives of the Stockholm Convention with regard to DDT include major shortcomings in periodic reporting by Parties to the Stockholm Convention, and deficiencies in reporting to the DDT Register.

**Conclusion:**

Global production and global use of DDT have shown a modest decline since the adoption of the Stockholm Convention.

## Background

The organochlorine dichlorodiphenyltrichloroethane (DDT) has been listed under the Stockholm Convention, with the main objective to protect human health and the environment from persistent organic pollutants (POPs) [1]. The Convention aims to reduce and ultimately eliminate DDT, but recognizes the acceptable production and use of DDT for disease vector control. DDT is one of the insecticides recommended by WHO for indoor residual spraying for malaria control [2].

Parties to the Convention (i.e. countries and other entities) can produce and use DDT for disease vector control in accordance with WHO recommendations and when locally safe, effective and affordable alternatives are not available. A DDT Register has been established under the Stockholm Convention in which countries are obliged to report their intention to produce or use DDT [3]. The continued need for DDT for disease vector control, which is subject to evaluation by the Conference of the Parties during its regular meetings held every 2 years, was confirmed in 2015.

To accelerate progress in achieving the Convention’s objective regarding DDT, a roadmap has been prepared to assist Parties with steps needed to make alternatives available for a sustainable transition away from DDT [4]. Moreover, in view of the continued need for DDT, a toolkit has been prepared to help DDT-using countries strengthen the sound management of DDT through its ‘life cycle’ stages [5].

A previous contribution reviewed the global status of DDT in 2007/08 [6]. As an update, the objective of the present paper is to evaluate trends in the production, use, export and import of DDT since adoption of the Stockholm Convention, over the period 2001–2014.

This paper was prepared in the context of an effectiveness evaluation undertaken pursuant to Article 16 of the Stockholm Convention [7]. The purpose of evaluation was to assess the impact of the Convention towards protecting human health and the environment from POPs. The Convention was adopted in 2001, went into force in 2004, and from 2004 onwards, a growing number of countries became Parties to the Convention. For this study, it was assumed that awareness among countries about the adverse health and environmental consequences of DDT, and the need for country-based action, was raised from the year of adoption (2001) onwards. This assumption is reflected in the period covered by our study.

The scope of this paper is to present changes in quantities in production and use of DDT, including progress in legal measures taken by countries to prohibit or restrict the production, use, import and export. Changes in DDT levels in humans and the environment have been reported elsewhere [8, 9]. As this is the first effectiveness evaluation cycle, it will provide a baseline for future evaluations. In addition, the time period covered by the evaluation allows for the study of trends.

## Methods

### Legal measures

Data on legal measures taken by countries in relation to DDT were obtained from official national reports for the Stockholm Convention, which Parties to the Stockholm Convention are obliged to complete and submit to the Secretariat of the Convention every 4 years. Three reporting cycles have been completed (Table 1); these national reports are in the public domain [10]. Forty-two Parties completed a single cycle, 38 Parties two cycles, and 28 Parties all three cycles; 108 Parties reported in at least one of the three cycles. This information implies that 42% of Parties to the Stockholm Convention (total Parties in 2014: 186) did not respond to any reporting cycle.

**Table 1:** Main data sources used for the analysis

Data source	Cycle	Reporting period	Responding Parties
National reports	1^a^	2003–06	45
	2^b^	2007–10	88
	3^c^	2011–14	69
DDT questionnaires	1	2000–02	21
	2	2003–05	12
	3	2006–08	31
	4	2009–11	23
	5	2012–14	30

Total number of Parties to the Stockholm Convention during each cycle of national reporting is indicated in the footnotes

^a^138 Parties in 2006

^b^179 Parties in 2010

^c^186 Parties in 2014

### Production, use, import and export

The primary source of data on production, use, import and export of DDT was the official country responses to the DDT questionnaire, which Parties to the Stockholm Convention and that produce or use DDT for control of malaria and other vector-borne diseases, are required to complete and submit once every 3 years [1, 11]. These data are managed by the Secretariat of the Stockholm Convention, and reviewed by the DDT Expert Group, as part of the evaluation of the continued need for DDT. Five cycles of questionnaire data were available for analysis (Table 1). DDT quantities are expressed as the amount of active ingredient (a.i.).

Production data were available from the DDT questionnaire from 2007 onwards (for China: from 2006 onwards). Production data prior to this year were obtained from project proposals submitted to the global environment facility, and workshop presentations by party delegates in the context of the Stockholm Convention [6]. In the case of the Democratic People’s Republic of Korea (DPR Korea), production data were available from the national implementation plan for the Stockholm Convention [12]. No production data were available for the years 2001, 2002 and 2004, apart from the data from DPR Korea.

Data on DDT use that were obtained from the DDT questionnaire showed various data gaps. Therefore, we supplemented the questionnaire data with data available from WHO for the period 2001–2009 [13]. Data on DDT use from DPR Korea were obtained from the national implementation plan for the Stockholm Convention [12]. For analysis of trends in global production and use since adoption of the Stockholm Convention, the available data were divided into two periods: 2001–2007 and 2008–2014 and compared the average annual data between the two periods.

Data on import and export of DDT were solely obtained from the DDT questionnaire.

## Results

### Legal measures

The majority of Parties to the Stockholm Convention reported having legal measures in place that prohibit, or restrict, the production, import, export and use of DDT (Table 2). The data show that 64% of 108 responding Parties reported having a prohibition of production on DDT; 74% with an import prohibition on DDT; and 82% with a prohibition on use of DDT in agriculture. Prohibition on DDT use in public health was less common (68%). Forty-one percent of Parties allowed restricted production and/or use of DDT for control of malaria and other vector-borne diseases.

**Table 2:** Adoption of legal measures regarding DDT

Legal measures on DDT	Total Parties		Parties, by year				
	Number	%	Before 2001	2001–2004	2005–2008	2009–2012	Year not indicated
Production prohibition	69	(64)	30	20	3	8	8
Import prohibition	80	(74)	28	23	6	8	15
Export prohibition	63	(58)	24	24	4	5	6
Prohibition on agriculture use	89	(82)	37	20	6	7	19
Prohibition on public health use	73	(68)	34	15	5	7	12
Restriction on production and/or use	44	(41)	8	17	8	1	10

Number (and %) of Parties that have adopted legal measures to control the production, import, export and use of DDT (out of 108 responding Parties). A sub-division is given to indicate the number of Parties according to the year of adoption or amendment of the legal measures

When comparing the situation before and after 2001, it is evident that major progress has been made in terms of countries adopting or amending their legal measures on DDT following adoption of the Stockholm Convention. Roughly half of the countries with legal measures in place have established or amended these measures after the year 2001. The Convention entered into force in May, 2004; from this year, Parties to the Convention were obliged to conform to it. Interestingly, the progress was most significant during the initial years after adoption of the Convention, 2001–2004. Of this progress, 35–67% was in 2001–2003. These observations confirm our assumption that compliance to the Convention began before Parties were obliged to conform to the Convention.

### DDT production

**Fig. 1:**
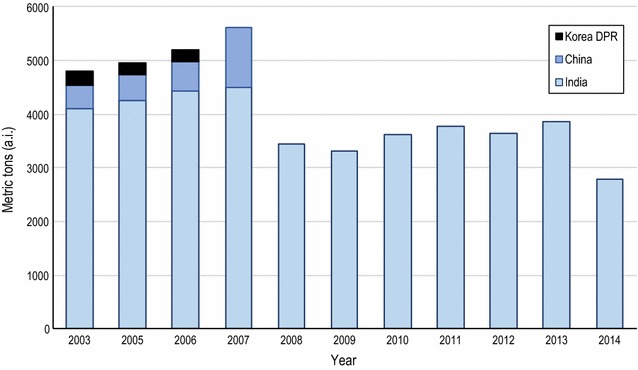
Annual global production of DDT. Data exclude DDT used as intermediate in the production of Dicofol, and use as additive in anti-fouling paints. Data for 2001, 2002 and 2004 are lacking

Since 2003, there have been three countries reporting production of DDT: India, China and the Democratic People’s Republic of Korea (DPR Korea). During the period 2003–2007, average production was 5144 metric tons per annum, but during the period 2008–2014, average production was 3491 metric tons p.a.—a decline of 32% during the 12-year period (Fig. 1). India has been the largest producer of DDT, with production still ongoing. China discontinued its production in 2008. Data from DPR Korea are only available until 2006 [12].

### DDT use

**Fig. 2:**
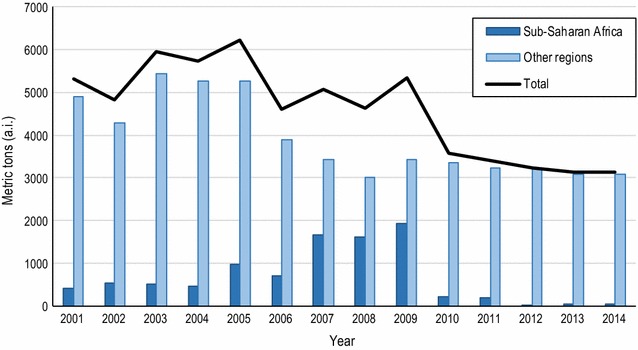
Annual global use of DDT. The contribution of sub-Saharan Africa and other regions is presented

Since adoption of the Stockholm Convention, DDT use showed a gradual decline from an average use of 5388 metric tons p.a. during the period 2001–2007 to 3772 metric tons p.a. during the period 2008–2014, which is a 30% decline over the 14-year period (Fig. 2). The decline was most evident after the Convention entered into force in 2004. All reported use of DDT was for public health, with the exception of DPR Korea where data indicated use in agriculture and forestry.

In sub-Saharan Africa, the use of DDT increased from 2001 until 2009, but declined thereafter. In this region, the increase in DDT use coincided with the time that major funding sources were mobilized to drive a massive scaling-up of vector control interventions, which included indoor residual spraying, aiming to control or eliminate malaria in countries of sub-Saharan Africa. This has increased the use of vector control insecticides, of which DDT constituted an important part [13]. After 2009, use of DDT declined when Ethiopia, Eritrea and Zambia switched to other insecticides, or other interventions, due to the development of DDT resistance, as reported in the DDT questionnaire and by several studies [14, 15].

DDT use by individual countries is presented in Table 3. Fourteen out of the 19 listed countries are in sub-Saharan Africa. Nevertheless, India stands out as the main user of DDT, responsible for 84% of global use over the period 2001–2014. Hence, the gradual decline in global use was to a large extent attributable to India.

**Table 3:** Annual use of DDT by selected individual countries

Country	Year													
	2001	2002	2003	2004	2005	2006	2007	2008	2009	2010	2011	2012	2013	2014
Botswana		^a^							*0*	*6*	*0*			^b^
Eritrea	*7*	*6*	*13*	8	12^a^	16	27	31	*10*	*14*	*17*	*0*	*0*	*0*
Ethiopia	*274*	*299*	*272* ^a^	311	327	406	*1200*	*1200*	*1350*	*0*	*0*	*0*		
Gambia						^a^			*17*	*11*	*11*			^b^
India	4893	4273	5243	5092	5092	3731^a^	*3413*	*3000*	*3415*	*3347*	*3223*	*3211*	*3091*	*3092*
Korea, DPR		^a^	190	179	166	160								
Madagascar	*0*	*60*	*40*	*30*	*0* ^a^	*0*	*0*	*0*	*0*	*0*	*0*	*0*	*0*	*0*
Mauritius	*1*	*1*	*1*	*1* ^a^	*1*	*1*	*2*	*3*	*0*	*1*	*0*	*0*	*0*	*0*
Morocco	*0*	*0*	*1*	*0* ^a^	*0*		*0*	*0*	*0*	*0*	*0*	*0*	*0*	*0*
Mozambique	0	0	0	0	366^a^	82	*201*	*143*	*300*	*150*	*98*	*0*	*0*	*12*
Myanmar	*8*	*1*	3	2^a^	2	1	0	0	0			*0*	*0*	*0*
Namibia	53	95	48	31	48^a^	61	88	23	92					^b^
Solomon Islands	2	1	0	1^a^	0	0	0	0	0					
South Africa	*11*	*5* ^a^	*54*	*62*	*65*	*75*	*73*	*59*	*64*	*16*	*47*	*24*	*30*	*18*
Sudan	*75*	*75*	*75*			^a^								
Swaziland	0	0	0	0	9	8^a^	7	7	*4*	*4*	*4*			^b^
Uganda	0	0	0	0^a^	0	*0*	*0*	*24*	*0*	*0*	*0*			
Zambia	0	0	0	11	42	0^a^	*23*	*33*	*24*	*19*	*0*	*0*	*0*	*0*
Zimbabwe	0	0	0	0	96	61	44	97	61			^a^		^b^

Use expressed in metric tons of active ingredient. Data in italic font are from the DDT questionnaires. Data in normal font are from WHO’s assessment [13], except for the data for DPR Korea which are taken from the national implementation plan for the Stockholm Convention [12]. Open spaces indicate missing data

^a^Year of ratification, acceptance, approval or accession of the Stockholm Convention

^b^Countries for which recent use of DDT has not been reported but indicated by independent data sources

In the most recent DDT questionnaire (reporting period: 2012–2014), only India, Mozambique and South Africa reported their use of DDT. In addition, independent publications indicate that five other countries, namely Botswana, Gambia, Namibia, Swaziland and Zimbabwe, have ongoing use of DDT for control or elimination of malaria [16–19]; these countries have not recently reported to the Secretariat of the Stockholm Convention. The combined use of these five countries is estimated at 150 metric tons p.a., based on data for the three most recently reported years for each country (i.e. 2009–2011 for Botswana, Gambia and Swaziland; and 2007–2009 for Namibia and Zimbabwe).

DPR Korea used DDT mainly in agriculture and forestry (91–97% of DDT used), which are not acceptable purposes under the Stockholm Convention; the remainder was used in public health [12]. No recent data on use are available from DPR Korea.

DDT has been reported for control of anopheline mosquito vectors of malaria and phlebotomine sandfly vectors of leishmaniasis. India has been using DDT both for control of malaria and leishmaniasis. Data from WHO indicate that over the period 2000–2009, 19% of the global use of DDT was for leishmaniasis control; the remaining 81% being for malaria control [20]. However, the DDT Expert Group reported that in recent years, India has substantially increased use of DDT for leishmaniasis control, whilst its use for malaria control declined. Consequently, in 2014, an estimated 42% of the global use of DDT was used for leishmaniasis control and 58% for malaria control [21].

Over the period 2003–2014, global annual use was 9% higher than global annual production of DDT, which suggests that some DDT was used from previous stocks.

### Import and export

During the period 2001–14, twelve African countries have reported on their import of DDT for use in their malaria control programmes. Up until 2009, imports originated predominantly from China, but after China discontinued its DDT production in 2008, it stopped its export in 2010. From then onwards, imports originated solely from India. South Africa has a formulation facility at which imported technical-grade DDT has been formulated and re-packaged for national use and for distribution to other African countries. Ethiopia also had a formulation facility for DDT, which was discontinued in 2009.

Inadequate data were available for a global assessment of obsolete stocks and waste of DDT or amounts that were sent for environmentally sound disposal.

## Discussion

The years following the adoption, and entry into force, of the Stockholm Convention have seen a modest decline in the global production and use of DDT. Evidently, the Stockholm Convention ‘tool’ has not been the only factor influencing this trend. Other contemporaneous factors which influenced DDT use included the up- or downscaling of operations of indoor residual spraying, and the detected levels of insecticide resistance.

In this respect, it is important to reiterate the developments that contributed to the acceptable purpose of continued use of DDT for disease vector control under the Convention. In 1996, South Africa had withdrawn DDT from its malaria control programme in Northern Kwazulu-Natal, switching to a pyrethroid insecticide instead, but this policy change had a negative outcome. Soon, malaria incidence rates increased sharply, and entomological investigations revealed that the culprit, a very efficient vector species, had re-invaded the country after DDT spraying had stopped and was highly resistant to the pyrethroid insecticides being used [22]. Reverting back to use of DDT was considered to be the only viable option. Other countries in the region were viewing the developments in South Africa; several countries were confronted with similar problems of pyrethroid resistance and, later on, they also reverted to the use of DDT. When from 2009 more African countries strengthened their capacity of insecticide resistance monitoring, high detected levels of DDT resistance in some countries lead to policy change and a substantial decline in DDT use in sub-Saharan Africa.

Insecticide resistance in malaria vectors against DDT and other recommended insecticides, particularly pyrethroids, is sweeping across Africa [19, 23]. This is reducing the choice of readily available insecticidal options for malaria vector control. In southern Africa, the main malaria vectors have become resistant to both pyrethroids and carbamates. In certain settings, this leaves only DDT and much more expensive organophosphates, such as pirimiphos-methyl, as immediate options for insecticidal control and for use in insecticide resistance management strategies [24].

Contrasting the irregular use of DDT across sub-Saharan Africa, the pattern in India has been relatively constant. India has used large quantities of DDT, to protect sizable human populations at risk of malaria and leishmaniasis with indoor residual spraying. It is significant that India has partly shifted its DDT use from malaria control towards leishmaniasis control—possibly in response to low DDT susceptibility in malaria vectors (DDT questionnaire responses of 2009, 2012). Further changes in DDT use could be foreseen in view of recent reports on high levels of DDT resistance detected in the sandfly vectors of leishmaniasis [25, 26]. Considering the size of vector control operations in India, any such changes will have a major impact on the global pattern of DDT use. Further potential shifts in the global use of DDT should be closely monitored, for example to target aedine mosquito vectors of arboviral diseases such as chikungunya, dengue and zika. The DDT Expert Group recommended that countries should seek WHO guidance before considering DDT for the control of vectors of arboviruses [27].

The experiences with insecticide resistance demonstrate the critical importance for countries to establish the technical capacity for monitoring of insecticide susceptibility, and for quality assurance of interventions, in order to facilitate timely and evidence-based decision-making on vector control [28]. More demanding, but also important, will be to study whether detected levels of resistance result in reduced effectiveness or control failure of interventions in terms of epidemiological impact on disease [29].

Readily available vector control methods that do not rely on chemical insecticides, such as house improvement and larval source management, deserve increased attention in future integrated vector management strategies [30–32], as do approaches to improve targeting of interventions in settings of pre-elimination of disease [33]. In addition, further support is needed for the development and evaluation of novel vector control tools, as alternatives to DDT, for which the road map is available [4].

The reported progress in countries across the globe in establishing or amending legal measures to prohibit or restrict DDT is encouraging, because it consolidates the path towards elimination of DDT use. Nevertheless, implementation of these legal measures will remain a challenge in many countries. WHO has highlighted critical shortcomings in how countries endemic with malaria and other major vector-borne diseases are regulating, managing and monitoring public health pesticides, which include DDT, particularly in WHO’s African Region [34, 35]. For example, illegal trade in DDT, and actual or suspected use of DDT outside of the health sector have been mentioned in the national reports by some countries.

Several limitations in implementation of the Stockholm Convention can be identified with regard to DDT, some of which may also apply to other POPs. The periodic national reporting to the Conference of the Parties has generally been deficient, with a low response rate and inaccurate or incomplete information contained in many submitted reports. In particular, the assessment and reporting on obsolete stocks, waste and disposal of DDT should be improved. Similarly, the response rate to the DDT Questionnaire has been inadequate, with several countries for which independent information sources indicate ongoing use of DDT failing to fulfill this specific requirement under the Convention [16–19]. There are indications that deficiencies in the quality of reporting (e.g. on dates, DDT amounts, and formulations) are an impediment for the comprehensive evaluation of the continued need for DDT by the Conference of Parties.

Moreover, there are deficiencies in the DDT Register. As of December 2016, seventeen Parties to the Convention were listed in the DDT Register, some of which current users of DDT. However, some Parties, which are known users of DDT, have not listed themselves in the DDT Register despite the obligation under the Convention. These shortcomings highlight the importance of establishing a quality assurance mechanism for reporting and a compliance mechanism, including non-compliance determination and possible measures in case of non-compliance.

## Conclusions

DDT continues to be used for control of malaria and leishmaniasis in accordance with the acceptable purpose under the Stockholm Convention. Following the adoption, and entry into force, of the Convention, there has been a modest decline in both global production and global use.

In some countries, DDT is used in response to the development of resistance of malaria vectors against pyrethroid and carbamate insecticides. Several other countries have switched to alternatives to DDT, in compliance to the Convention, or, after resistance monitoring demonstrated high levels of DDT resistance. This has contributed to the modest decline in the global use of DDT. The declining trend is expected to continue in the years ahead in view of recent data on DDT resistance in vectors of malaria and leishmaniasis.

Major progress has been made by countries establishing or amending their legal measures on DDT since the Stockholm Convention was adopted in 2001. These developments, together with instruments such as the roadmap on development of alternatives of DDT [4], consolidates the path towards elimination of use of DDT. The majority of countries reported that they have legal measures in place that prohibit, or restrict, the production, import, export and use of DDT.

Implementation of the Stockholm Convention with regard to DDT, is constrained by major shortcomings in the national reporting, DDT questionnaire responses, and DDT Register. This calls for quality assurance of reporting and a compliance mechanism. Data on obsolete stocks, waste and disposal of DDT require particular attention.

## Abbreviations

DDT: dichlorodiphenyltrichloroethane
WHO: World Health Organization
DPR: Korea Democratic People’s Republic of Korea
